# Prevalence of Nitroimidazole-Refractory Giardiasis Acquired in Different World Regions, Sweden, 2008–2020

**DOI:** 10.3201/eid3106.250076

**Published:** 2025-06

**Authors:** Karin A. Ydsten, Joanna Nederby Öhd, Urban Hellgren, Hilmir Asgeirsson

**Affiliations:** Capio St. Görans Hospital, Stockholm, Sweden (K.A. Ydsten); Karolinska University Hospital, Stockholm (K.A. Ydsten, U. Hellgren, H. Asgeirsson); Region Stockholm, Stockholm (J.N. Öhd); Karolinska Institutet, Stockholm (U. Hellgren, H. Asgeirsson)

**Keywords:** giardiasis, parasites, Giardia, nitroimidazole, treatment, refractory, antimicrobial resistance, epidemiology, prevalence, India, Sweden

## Abstract

Treatment-refractory giardiasis is an emerging clinical problem. Of 4,285 giardiasis cases identified during 2008–2020 in Stockholm, Sweden, 102 (2.4%) were nitroimidazole refractory. Among cases acquired in India, the percentage was high (64/545 [12%]) and increased over time. The region of acquisition needs to be taken into consideration when managing patients.

*Giardia intestinalis* (*G. lamblia, G. duodenalis*) is an intestinal protozoal pathogen found worldwide; the highest incidence occurs in developing countries. For decades, the first-line treatment for giardiasis has been the 5-nitroimidazoles, such as metronidazole and tinidazole (*1*). Since the early 2000s, reports of giardiasis refractory (resistant) to 5-nitroimidazoles have increased, posing a clinical challenge. The reports are mostly from travelers returning from the Indian subcontinent (*2*–*6*). Information on the prevalence of giardiasis refractory to 5-nitroimidazoles in India and global epidemiology is scarce. To examine global prevalence, we assessed the percentage of nitroimidazole-refractory disease among giardiasis cases acquired in different geographic world regions.

## The Study

In Sweden, giardiasis is a notifiable disease. We retrospectively extracted cases reported during January 2008–December 2020 from Stockholm County with information on age, sex, and country of infection from the national electronic notification system (SmiNet). In Stockholm, giardiasis patients not responding to treatment (repeat fecal sample testing is generally only done if symptoms continue) are referred to specialized adult and pediatric centers at Karolinska University Hospital, which are the only clinics providing second-line treatment alternatives because those require special prescribing licenses. We retrospectively evaluated medical records of all giardiasis cases seen at the centers during the study period. We defined a case as nitroimidazole refractory if the case had a positive fecal sample for giardiasis by microscopy (whole study period) or PCR (from 2016) after a full 5-nitroimidazole treatment course (metronidazole 400 mg 3×/d for 5–7 days or a single dose of tinidazole 2 g) and no indication of reinfection. The treating clinician determined whether the case was related to travel. The Regional Ethical Review Board in Stockholm (approval nos. 2018/2309-31 and 2020-06183) approved the study.

During the 13-year study period, Stockholm had 4,285 notified giardiasis cases, of which 3,172 (74%) were acquired abroad and 881 (21%) were acquired domestically; 232 had unknown acquisition (Table). The mean incidence of all cases was 15.2/100,000 population/year and incidence among domestic cases was 3.2/100,000 population/year. 

**Table:** Giardiasis by country of acquisition and rate of nitroimidazole-refractory cases, Stockholm, Sweden, 2008–2020

Origin of infection*	Total no. cases	No. (%) nitroimidazole-refractory cases
Asia	1,337	73 (5.5)
India	545	64 (11.7)†
Thailand	168	4 (2.4)
Pakistan	94	1 (1.1)
Iraq	71	0
Turkey	65	0
Afghanistan	58	0
Syria	45	0
China	36	0
Sri Lanka	33	0
Cambodia	25	0
Indonesia	25	0
Bangladesh	23	2 (8.7)
Nepal	7	2 (28.6)
Africa	1,115	17‡ (1.5)
Tanzania	136	3 (2.2)
Somalia	133	1 (0.8)
Ethiopia	105	1 (0.9)
Kenya	94	0
Eritrea	93	0
Uganda	65	2 (3.1)
Egypt	57	0
Gambia	45	0
Sudan	37	0
South Africa	26	1 (3.8)
Morocco	23	0
Americas	349	1 (0.3)
Colombia	67	1 (1.5)
Brazil	55	0
Peru	36	0
Mexico	28	0
United States	23	0
Europe	1,247	11§ (0.9)
Sweden	881	5 (0.6)
Spain	107	2 (1.9)
Greece	36	0
France	29	1 (3.4)
Oceania	5	0
Unknown	232	0
Total	4,285	102 (2.4)

*Countries with >20 giardiasis cases in total are shown, as well as countries with >2 treatment refractory cases. †p<0.0001 for India compared with rest of Asia and for India compared with the rest of the world (by χ^2^ test). ‡In addition, the following countries had 1 case: Angola, Benin, Central African Republic, Democratic Republic of the Congo, Ghana, Madagascar, Sudan, Chad, and Zimbabwe. §In addition, the following countries had 1 case: Belgium, Italy, and Switzerland.

The number of cases identified as nitroimidazole refractory was 102 (2.4%), and 97/102 (95%) were related to travel. Of 102 treatment-refractory cases, 96 had received >2 courses of 5-nitroimidazoles. In the first half of the study period (January 2008–June 2014), the percentage of nitroimidazole-refractory cases was 2.0% (46/2,255), compared with 2.8% (56/2,030) in the second half (July 2014–December 2020; p = 0.12). For cases acquired specifically in India, the percentage rose from 8.5% (29/341) to 17.2% (35/204) between the 2 periods (p = 0.002 by χ^2^ test). 

The median patient age was 30 (range 0–89) years in the total study population and 35 (range 1–78) years in patients with refractory disease. Among 4,285 cases, 55% were in male patients and 45% were in female patients; slightly more (55/102) male patients had nitroimidazole-refractory infections than female patients (47/102). Only 2 of the nitroimidazole-refractory cases were identified with immunosuppressive conditions, 1 receiving rituximab and methotrexate and 1 prednisolone; none had other immunoglobulin deficiencies or HIV. The treatment and outcome of the patients are described elsewhere (*6*).

The percentage of patients with nitroimidazole-refractory disease varied by countries visited (Table). A high prevalence of 12% (64/545) was seen in persons returning from India, compared with 1.1% (9/792) from the rest of Asia (excluding India; p<0.0001) and 1.0% (38/3,740) from the rest of the world (p<0.0001): Asia (1.1%; 9/792), Africa (1.5%; 17/1,115), Europe (0.9%; 11/1,247), and the Americas (0.3%; 1/349). The highest (29%; 2/7) prevalence was noted in patients infected in Nepal. Among 881 cases acquired domestically in Sweden, 5 (0.6%) were nitroimidazole refractory. Only 1 of those 5 case-patients had known contact with a travel-related case. Among the 97 travel-related nitroimidazole-refractory giardiasis cases (all symptomatic <28 days of returning from travel), tourism was the most common reason for travel (75%; 73/97), followed by work or studies (11%; 11/97), immigration (9%; 9/97), and visiting friends or relatives (4%; 4/97). 

This study conducted in a low-endemic population-based setting showed that the proportion of nitroimidazole-refractory giardiasis was >10 times higher in persons who had acquired the infection in India (12%) compared with other parts of the world (1.0%). Of note, the proportion of nitroimidazole-refractory disease among cases acquired in India rose over the study period (from 8.5% to 17%). The percentage of nitroimidazole-refractory cases acquired in India (12%) was similar to that found in a previous small study from India (10%; 8/82) (*7*). Other studies from specialized centers in Europe have reported substantially higher percentages from India, up to 50% (*2*,*3*,*6*). However, those studies were small and might have been biased because they mostly included referred patients, whereas this study is based on all giardiasis cases identified in a large well-defined geographic area. Data from countries outside Asia are sparse in the literature. Studies from Cuba have reported a metronidazole refractory rate of 46% (248/456) in 2017–2018 and 15% (11/75) in 2009 (*8*,*9*). That rate is much higher than the 0.3% (1/349) noted from the Americas in this study, but that total included only 14 cases from Cuba (none treatment refractory).

The reason for the higher, and increasing, prevalence of nitroimidazole-refractory disease in India compared with other countries is not known. High drug pressure (frequent use of 5-nitroimidazoles) could perhaps be an explanation. That pressure is seen with bacterial drug resistance, also common in India (*4*,*10*). The lower prevalence found in neighboring Pakistan (1%), which has similar living conditions, is at the same time difficult to explain. To understand those geographic and temporal variations, better knowledge on the mechanisms behind nitroimidazole-refractory giardiasis is needed.

Treatment-refractory disease with persistence of protozoa in fecal samples may result from parasite drug resistance, but host-related factors, such as immunoglobulin deficiencies and HIV, can also play a role (*11*). Drug resistance is difficult to evaluate in clinical settings because antimicrobial drug susceptibility testing for *Giardia* is lacking (*12*,*13*), and the underlying molecular resistance mechanisms are not well understood (*1*,*14*). In our cohort, only a small percentage (2%) of patients with nitroimidazole-refractory diseases had known immunosuppressive conditions. Furthermore, all patients (n = 56) who were treated with quinacrine improved (*6*). Although quinacrine has been reported to have good efficacy against nitroimidazole-refractory giardiasis (clinical cure rates of 98% and parasitologic cure rates of 89% from pooled studies) (*5*,*6*,*15*), increased use might pose a risk for further resistance. Without reliable diagnostic assays on antimicrobial drug resistance, the management of treatment-refractory giardiasis will remain a clinical challenge.

A limitation of the study is that some treatment-refractory cases might not have been referred to our center and thereby missed. Missing cases would lead to an underestimation of nitroimidazole-refractory disease, but the underestimation would most likely be similar for different regions. Similarly, the study focused on clinically refractory disease, and asymptomatic carriage after nitroimidazole treatment could go unnoticed. Another limitation is that, despite a large total number of cases, the numbers from specific countries were often low, which makes drawing conclusions on a country level difficult. Strengths of the study are its population-based approach, the low likelihood of missed cases because in Sweden giardiasis is notifiable, the low-endemic setting with small risk for reinfections, and that all nitroimidazole-refractory cases were well defined with a thorough follow-up.

## Conclusions

The results of this study show that nitroimidazole-refractory giardiasis is prevalent on the Indian subcontinent but is substantially less common in the rest of the world. Further studies on the epidemiology of treatment-refractory giardiasis and possible underlying resistance mechanisms are needed. Clinicians should take into consideration the region where infection was acquired when managing and treating giardiasis patients.
